# Research on Flower Pattern and Roll Positioning Optimization for Roll Forming Process of TA4 Coiled Tubing

**DOI:** 10.3390/ma17246164

**Published:** 2024-12-17

**Authors:** Xuwen Deng, Songxiao Hui, Xiongyue Ye, Wenjun Ye, Yang Yu, Yunbo Li

**Affiliations:** 1 GRINM (Guangdong) Institute for Advanced Materials and Technology, Foshan 528051, China; dengxuwen@grinm.com; 2 GRIMAT Engineering Institute Co., Ltd., Beijing 101407, China; wenjun_ye@sina.com (W.Y.); yuyang@grinm.com (Y.Y.); liyunbo0111@126.com (Y.L.); 3 Guangdong ForeWeld Corporation Limited, Guangzhou 510700, China; 4 State Key Laboratory of Nonferrous Metals and Processes, China GRINM Group Co., Ltd., Beijing 100088, China; 5 Key Laboratory for Advanced Materials Processing (MOE), Institute for Advanced Materials and Technology, University of Science and Technology Beijing, Beijing 100083, China; 6 General Research Institute for Nonferrous Metals, Beijing 100088, China

**Keywords:** TA4 titanium, coiled tubing, roll forming, flower pattern, roll positioning

## Abstract

Titanium alloy coiled tubing (CT) has great superiority while operating in deep wells with severe conditions, but it faces serious challenges during roll forming like high springback, edge cracking and surface wearing. In order to utilize the high precision and high efficiency production of titanium alloy CT, it is urgently necessary to develop a detailed process design. Based on the W-forming strategy and the cubic curve of the strip edge projection, the flower pattern and roll design for the 21-pass roll forming process were completed. Different tube dimensions and roll positionings were researched regarding the aspects of contact state, stress–strain distribution and geometry ovality using numerical simulation. The thickness/diameter ratio of the tube that can be formed should be within an effective range: exceeding the range leads to edge cracking, and an insufficient ratio leads to high springback. Roll positioning significantly affects the contact state and stress distribution, but leads to no major differences in the tube geometry. Finally, the trial production of the TA4 titanium CT with a size of Φ50.8 × 4 mm was completed successfully and was consistent with the simulation results.

## 1. Introduction

Coiled tubing (CT) is the term given to a non-threaded tube manufactured by a metal strip continuously undergoing multi-pass transverse bending and subsequent closure by welding. When not in use, CT is wrapped on a reel, can be run to operate a well directly through straightening–bending–straightening deformation and is then collected again on the reel after finishing the operation [1]. CT has been widely used in various aspects of oil and gas exploitation and has the advantages of safe well intervention, less tripping time and an efficient operation cycle [2,3].

Generally, with increasing depths being exploited through drilling, the working conditions of CT tend to be harsh, as it is confronted with high temperatures, high pressures and serious corrosion [4]. Traditional steel CT has gradually become unsuitable for use in extreme conditions. Titanium alloy has the advantages of high specific strength, superior performance in high temperatures and excellent corrosion resistance and is the perfect substitute material for CT [5]. However, due to its inherent properties of a high strength and low elastic modulus, the roll forming process faces significant difficulties, including high springback, narrow effective forming range and wearing with rolls. The forming process on the production line mainly depends on the experience of workers, for whom there is a lack of data and theory to use for guidance.

For the research method of the metal roll forming process, early research focused on theoretical analysis and later developed into finite element numerical simulation. Panton et al. [6,7] divided the roll forming process between two adjacent passes into three regions. Based on this, the deformation forms in roll forming were classified into four types—longitudinal stretching, longitudinal bending, transverse bending and shear—and given the expressions of strain and plastic work for each deformation form. Bhattacharyya et al. [8], using the minimum deformation energy method, obtained the expression of deformation length of flange length, channel angle and thickness, and the theoretical results were verified by roll forming experiments using mild steel and aluminum strips. Farzin et al. [9] introduced the buckling limit of strain (BLS), which is the maximum formable strain of a single pass roll to avoid edge buckling, for the first time. BLS could be conveniently calculated according to the materials’ properties and numerical simulation in order to guide the design of the flower pattern. Kiuchi et al. [10] clarified the deformation characteristics of electric resistance welded (ERW) pipes during each forming stage affected by the factors of roll geometry and position, product size and mechanical properties, considering the mathematical model and numerical simulation model comprehensively. Kim et al. [11] examined problems in thick strip roll forming, such as edge thickness decreases, edge forming deficiency and large differences in perimeter, and the initial strip edge shape prediction program was developed for simulation. The results show that the inverted triangle is the optimal edge shape in the double radius forming strategy. Meng et al. [12] proposed a theoretical method for predicting edge buckling in roll forming based on the average longitudinal strain, and it was further verified by the finite element software ABAQUS. Samusev et al. [13] analyzed the advantages and disadvantages of single radius and double radius forming strategies for small–medium diameter tubes, concluding that the double radius forming process with a downhill method was the most suitable. Suckow et al. [14] analyzed the effects of a double radius forming strategy and two W-forming strategies on the formation of 7075 aluminum tubes; different strategies have different strain distributions and buckling rates, even though the geometric shapes are the same. In addition, the boundary condition constraint was used to simplify the welding process to obtain the optimal welding pass roll arrangement. Egger et al. [15] established a fully multi-field coupling simulation model consisting of roll forming and high-frequency induction welding for steel 34MnB5 tubes. The model accurately predicted the geometry size, residual stress, temperature distribution and microstructure evolution of the tube and was able to investigate the complex physical interactions during manufacturing and high frequency induction welding.

In addition to the traditional roll forming for tubes, the process of flexible roll forming has since evolved. Cheng et al. [16] modified the rolls of one to five passes into flexible rolls with an involute contour and designed the forming process of EWR tubes of seven sizes. The results show that the uniformity of the transverse strain distribution was improved, and the tube produced by flexible rolls was able to reduce the overall weight of rolls by about 35% while meeting the accuracy requirements. Hu et al. [17] analyzed the effective plastic strain distribution of high frequency welded pipes during cage roll forming; the maximum plastic strain was concentrated at the center of the strip due to the uneven downhill amount. Jiang and Chen et al. [18,19] discovered the “non-bending area” range at different cage roll forming stands. Based on the relevant parameters of the non-bending area—for instance, the relative positions and relative curvature—the flower pattern of cage roll forming can be designed. Then, given the geometric constraint between the strip and rolls, combined with the geometric constraint and flower pattern, the final roll positioning parameters were determined. Kasaei et al. [20,21,22,23] assessed the difference in edge buckling between traditional roll forming and cage roll forming and found that the low ratio of thickness/diameter and wide strip width are more likely to cause edge buckling in the cage roll forming process; if they exceed a certain limit, edge buckling will inevitably occur.

The above studies show that the roll forming process method and theory of steel tubes are now relatively well-developed, and the relevant specification has been formulated [24], but research on the process of titanium alloy formation is scarce. Only Badr et al. [25,26] studied the V-shaped roll forming of a high strength Ti-6Al-4V titanium alloy, and the results indicate that the traditional isotropic hardening model obviously overestimated the springback rate. They proposed a new constitutive model based on the homogeneous anisotropic hardening approach to characterize the forming behavior during cold roll forming, and thus revealed the mechanism of low springback. Yue et al. [27] investigated the influence of frame spacing, forming speed, roll gap and downhill level on the edge wave during the roll forming process of the TA2 tube using an orthogonal test. The downhill level was found to be the most important parameter affecting the edge wave, and the optimal forming parameters were determined.

In this study, to enable CTs to cope with their harsh working conditions and to improve their service life, we selected TA4, the highest grade of industrial pure titanium, which offers good corrosion resistance, machinability and weldability, to replace traditional steel CTs and conduct research into roll forming processes. Based on the W-forming strategy, the arrangement and positioning of 21 passes rolls were determined, and the flower pattern was designed according to the cubic curve of the strip edge projection. Then, combining the isotropic material model of the TA4 strip with the geometric model of rolls, the numerical simulation model for roll forming process was established. The influence of different tube dimensions and roll positioning on springback, stress–strain distribution and contact state during roll forming was analyzed, the forming defects were reasonably predicted and the process parameters were optimized. Finally, guided by the research results, a defect-free trial example of a TA4 tube with a size of Φ50.8 × 4 mm was successfully created on the production line.

## 2. Numerical Simulation Modeling Method for Roll Forming

### 2.1. Tensile Test and Material Modeling

**Figure 1:**
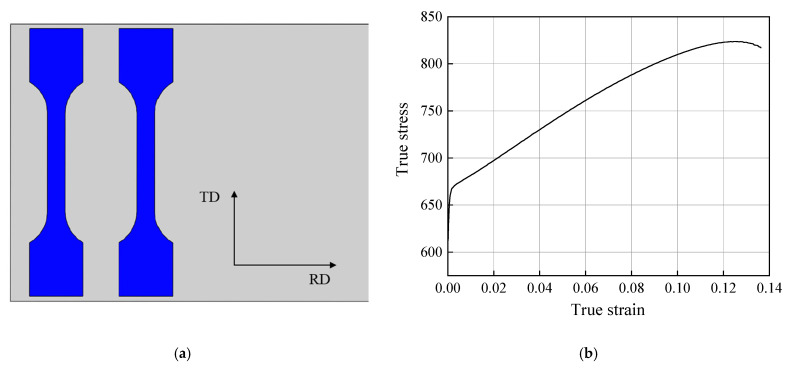
Tensile tests of the strip: (**a**) sampling diagram; (**b**) flow stress curve.

The material used in the experiment was a TA4 titanium strip with a thickness of 4 mm and a width of 149 mm. The tensile specimens were cut along the transverse direction of the strip, as shown in Figure 1a, and subjected to uniaxial tensile testing using an MTS universal tensile testing machine, following the standard GB/T228.1-2010. Figure 1b presents the flow stress curve in the plastic stage, which can be characterized by a typical strain hardening model, and when the plastic strain increases to 0.125, the stress reaches its maximum; this is then followed by necking until fracture. Based on the flow stress curve, an isotropic Johnson–Cook constitutive model was established and is expressed in Equation (1). The material properties are summarized in Table 1.

(1) $$\sigma =612+584{\varepsilon_{p}}^{0.5}$$

### 2.2. Roll Arrangement and Positioning

There are three commonly used forming strategies for tubes: single radius-forming, double radius-forming and W-forming [28]. Of these, the W-forming method has proven to be the most appropriate strategy for high-strength material or thick tubing, and can effectively reduce the edge height during first pass, avoid edge defects and improve the welding quality [14,16].

**Figure 2:**
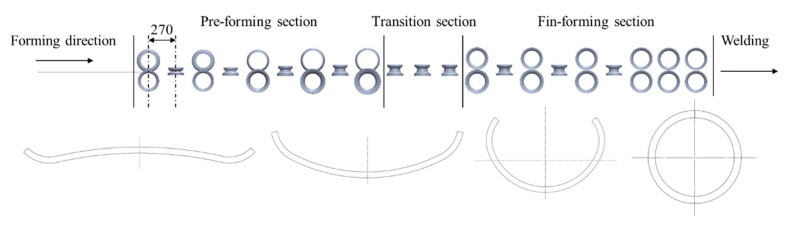
Roll arrangement in the roll forming process.

In this work, twenty-one sets of forming stations, including eleven pairs of horizontal rolls and ten pairs of vertical rolls, were used for the roll forming process with the W-forming strategy. The distance between adjacent forming stations is 270 mm. The forming process consists of three stages as follows: the pre-forming section, the transition section, and the fin-forming section. In the pre-forming stage, the edge region is formed to target the size in the first pass through the W shape, and subsequent passes adopt double-radius U-shaped forming. After pre-forming, the strip transits three passes of vertical rolls, and the cross-section transitions from a U shape to a C shape. Finally, the strip is gradually converged to a circle and is formed into a tube in the closed pass [29]. The roll arrangement is shown in Figure 2.

**Figure 3:**
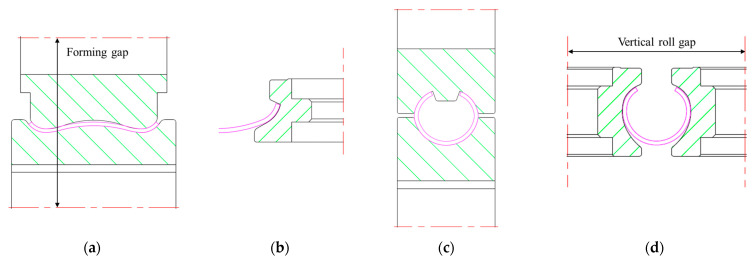
The assembly between the strip and rolls in different forming stages: (**a**) pre-forming horizontal rolls; (**b**) pre-forming vertical rolls; (**c**) fin-forming horizontal rolls; (**d**) fin-forming vertical rolls.

Based on the abovementioned roll arrangement, the strip and roll positioning schematic diagram of each forming stage is illustrated in Figure 3. The positioning of the pre-forming vertical roll (Figure 3b) and the rounding horizontal roll (Figure 3c) are matched with the cross-section of the strip, which does not need to be adjusted. The experiment only needs to adjust the distance between the upper and lower rolls in Figure 3a to control the forming gap as well as the spacing between two vertical rolls in Figure 3d to control the looseness or tightness of the rounding. The roll positioning standards are as follows: when the distance between the upper and lower rolls is equal to the thickness of the strip, the forming gap is 0 mm; when the double radius arc tangent point of vertical roll and the strip coincide, the vertical roll gap is 0 mm.

### 2.3. Geometric Design of Flower Pattern

**Figure 4:**
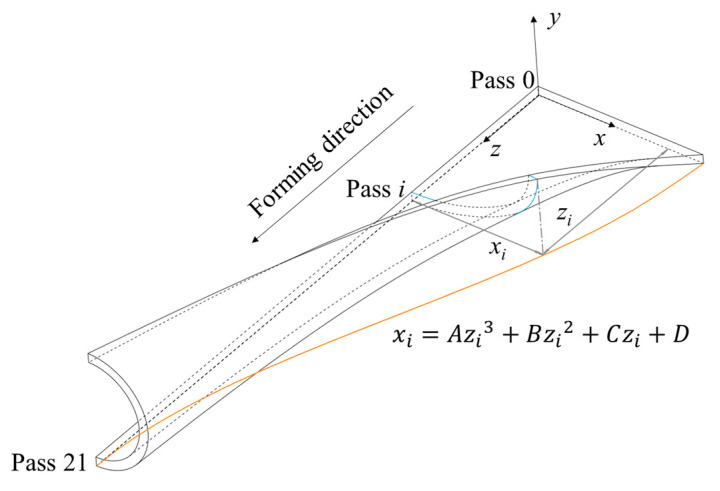
The edge function for roll forming.

According to the research of Ona et al. [30], the projection curve of the strip edge along the y-axis on the x–z plane can generally be taken as a cubic function, as shown in Figure 4.

Where *z_i_* is the longitudinal forming distance when the strip passes through the *i*th rolls, *x_i_* is projection distance on pass *i*, and *A*–*D* are the parameters to be determined.

The boundary conditions are as follows:

(2) $$\left\{\begin{array}{c} x_{0}=L/2\\ x_{21}=0\\ \frac{dx}{dz}{|}_{i=0,i=21}=0 \end{array}\right.$$

Substitute boundary conditions into the cubic function, and the projection curve *x_i_* can be obtained as follows:

(3) $$x_{i}=\frac{L}{2}\left[{2\left(\frac{i}{21}\right)}^{3}-3{\left(\frac{i}{21}\right)}^{2}+1\right]$$

**Figure 5:**
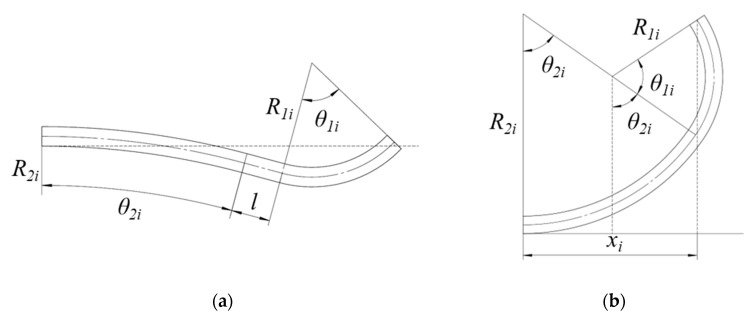
Cross-section of W-forming process: (**a**) first and second passes; (**b**) the other passes.

In addition, according to geometric relationships presented in Figure 5, the following equation can be obtained:

(4) $$\;x_{i}=\left\{\begin{array}{l} R_{2i}sin\theta_{2i}+lcos\theta_{2i}+R_{1i}\left[\sin\theta_{1i}+\sin\left(\theta_{1i}\;-\theta_{2i}\right)\right]\;\;W\;shape\\ {(R}_{2i}\;-R_{1i})sin\theta_{2i}+R_{1i}sin\left(\theta_{1i}+\theta_{2i}\right)\;\;\;\;\;\;\;\;\;\;U\;shape \end{array}\right.$$

And the length of the neutral axis is constant, we can obtain

(5) $$R_{1i}\theta_{1i}+R_{2i}\theta_{2i}+l=L/2$$

**Figure 6:**
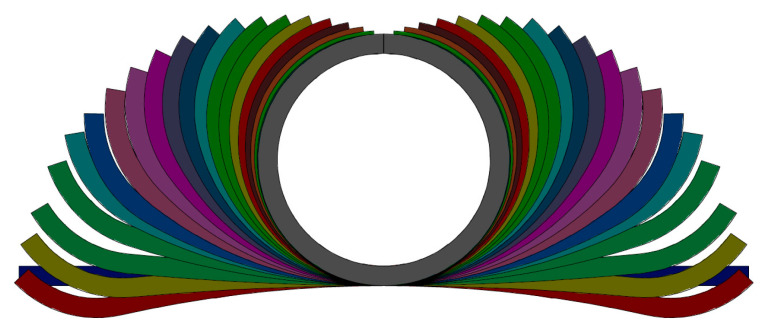
The flower pattern of TA4 tube with a size of Φ50.8 × 4 mm.

When combining Equations (3)–(5) and setting a value of *θ*_1_, the unique shape and cross size of each pass can be determined. The ultimate flower pattern is shown in Figure 6, and the detailed dimensions of each pass are listed in Table 2.

### 2.4. Numerical Simulation Details

The numerical simulation model of roll forming process was established using ABAQUS software, with the dynamic–explicit method for simulation. The model consists of 43 parts, including 21 pairs of rolls and a strip, in which the rolls are discrete 3D shell rigids and the strip is a deformable body. The friction coefficient between the strip and rolls was set to 0.1. The strip moves at a linear velocity of 500 mm/s along the +Z direction. The positions of all rolls are fixed, where the horizontal rolls act as forming rolls, rotating around the central axis at a speed of 5.26 rad/s, corresponding to the linear velocity of the strip; the vertical rolls are guiding rolls and do not rotate actively, and only the degree of freedom of UR2 is trailing movable.

**Figure 7:**
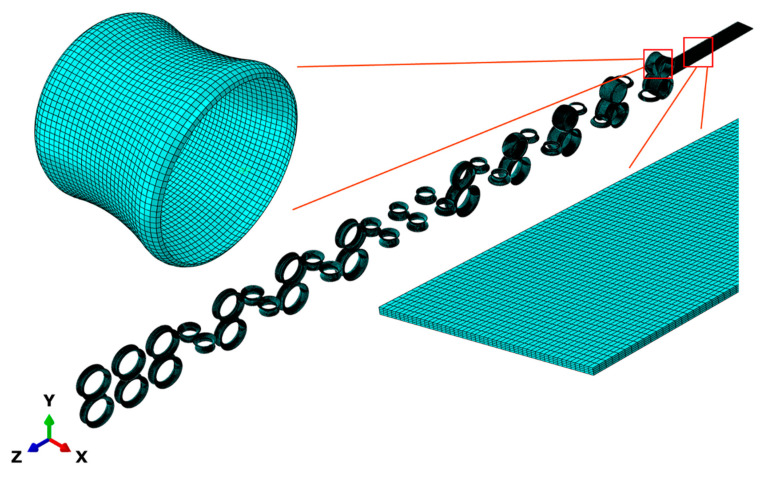
The mesh model of roll forming.

The rolls were meshed using R3D4 elements with a size of 5 mm. The strip was meshed with an eight-node-reduced integration element (C3D8R). The longitudinal and transverse mesh sizes of the strip were set to 10 mm and 3 mm, respectively, and four layers were used in the thickness direction. The meshed model is presented in Figure 7.

The mass scaling factor was set as 3000 to accelerate computation efficiency. For the whole model, the ratio of kinetic energy (ALLKE)/internal energy (ALLIE) is 0.026, and the ratio of artificial strain energy (ALLAE)/internal energy (ALLIE) is 0.085. These results show that using dynamic–explicit method to simulate roll forming process is reliable, and no significant hourglass phenomenon occurred in the reduced integration elements. Meanwhile, the computing time was 32 h, which is also acceptable.

### 2.5. Simulation Experiment Scheme

**Figure 8:**
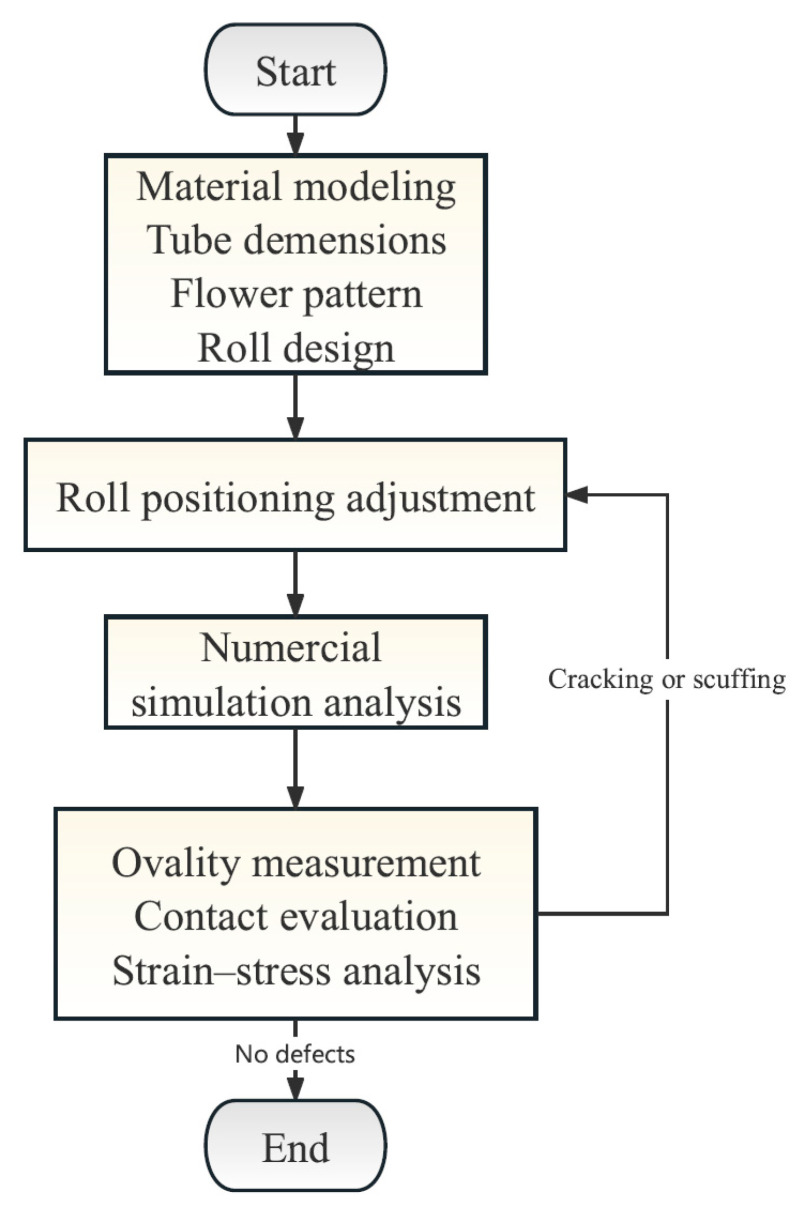
The optimization flowchart for the roll forming process.

Figure 8 presents the flowchart for optimizing the roll forming process. Under the premise of determining the flower pattern and roll sizes, the ovality, strain–stress distribution and contact condition were evaluated by adjusting the roll positions to ensure forming accuracy and to minimize defects. The numerical simulation model established in this paper explores the influence of tube dimensions (different thickness) and roll positioning (forming gap and vertical roll gap, as shown in Figure 3) on the roll forming process. The specific simulation schemes are listed in Table 3.

## 3. Results and Discussion

### 3.1. Effect of Tube Thickness

**Figure 9:**
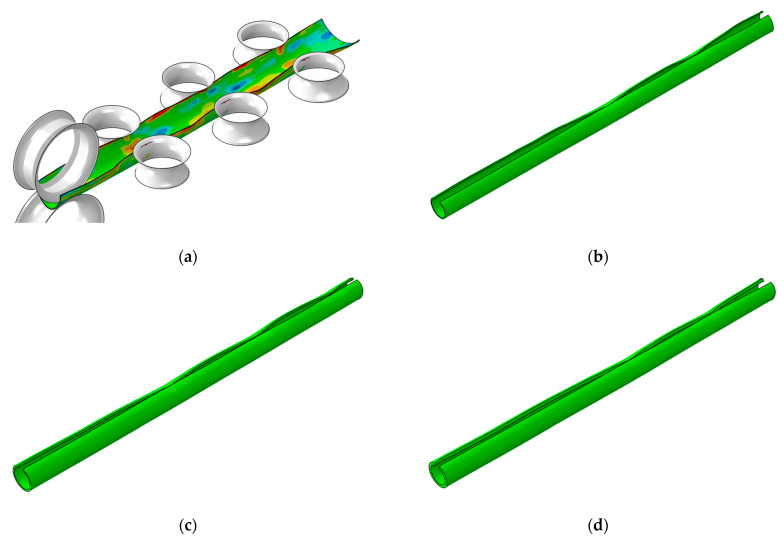
Numerical simulation result of tubes with different thickness: (**a**) 2 mm; (**b**) 3 mm; (**c**) 4 mm; (**d**) 6 mm.

The final schematic diagram of the tubes, formed with varying thickness specifications, is shown in Figure 9. It can be seen that the strip successfully forms into a tube without obvious defects when the thickness ranges from 3 to 6 mm. The springback gap increases as the thickness decreases. However, the strip with a thickness of 2 mm fails to bite into the rolls during the 13th pass due to its excessive springback, which exceeds the roll contour.

**Figure 10:**
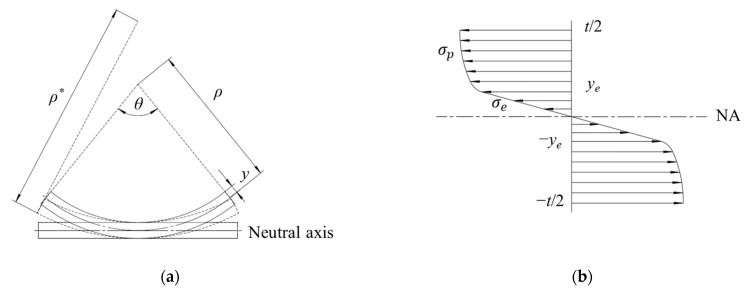
Representation of traverse bending: (**a**) bending and springback; (**b**) stress distribution across the thickness.

Figure 10 shows the schematic diagram of transverse bending which demonstrates the mathematical calculation of strip bending and springback. For the layer at a distance *y* above the neutral axis, the strain can be described as follows:

(6) $$\varepsilon =\frac{∆L}{L}=\frac{\rho \theta -\left(\rho -y\right)\theta }{\rho \theta }=\frac{y}{\rho }$$

The stress distribution across the thickness after bending is shown in Figure 10b. Taking *y_e_* as the interface, the stress in the elastic region and the elastic–plastic region of the TA4 strip can be formulated as follows:

(7) $$\left\{\begin{array}{c} \sigma_{e}=E\cdot \frac{y}{\rho }\;\;\;\;\;\;\;\;\;\;\;0<y\leq y_{e}\\ \sigma_{p}=612+584{\left(\frac{y-y_{e}}{\rho }\right)}^{0.5}\;\;\;\;\;y_{e}<y\leq \frac{t}{2} \end{array}\right.$$

The total bending moment can be expressed as follows:

(8) $$M=2\left[\int_{0}^{y_{e}}\sigma_{e}ydy+\int_{y_{e}}^{\frac{t}{2}}\sigma_{p}ydy\right]$$

After unloading, elastic recovery occurs in the strip and the bending radius changes from *ρ* to ρ*. The springback ratio is given as follows [31]:

(9) $$\frac{\rho *}{\rho }=\frac{\rho M}{\mathrm{EI}-\rho M}$$

**Figure 11:**
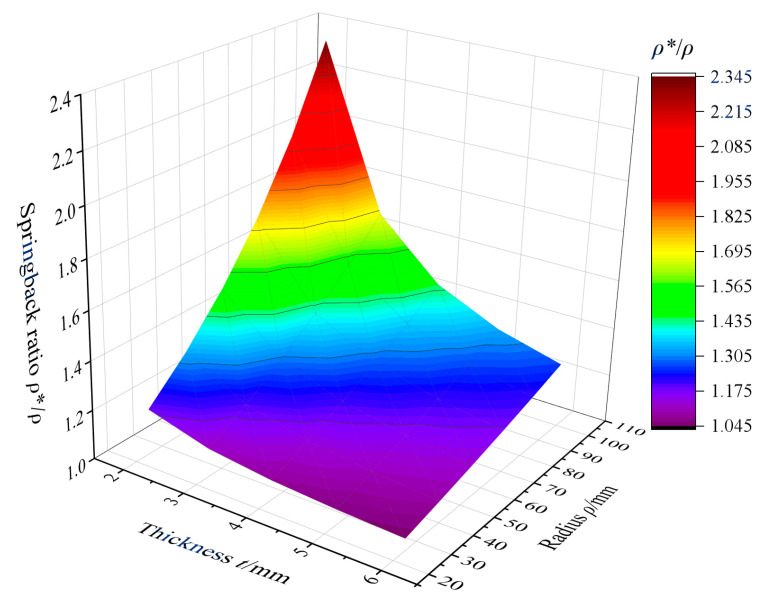
The relationship between the springback ratio, springback radius, and thickness.

Combining Equations (7)–(9), the function of the springback ratio regarding bending radius and thickness can be calculated, and the relationship is shown in Figure 11. The results indicate that the springback ratio increases with decreasing strip thickness and the increasing bending radius. For a bending radius of 25.4 mm, the corresponding springback ratios for strips of 2, 3, 4, and 6 mm thickness are 1.19, 1.11, 1.08, 1.05, respectively. The springback ratio increases significantly when the thickness is reduced from 3 mm to 2 mm. This is due to the decrease in the total strain. Thus, the proportion of the elastic range (*y_e_*/*t*) increases significantly, which exceeds the allowable springback forming limit. Taking a springback ratio of 1.15 as the critical value, the corresponding minimum formable thickness is determined to be 2.6 mm.

**Figure 12:**
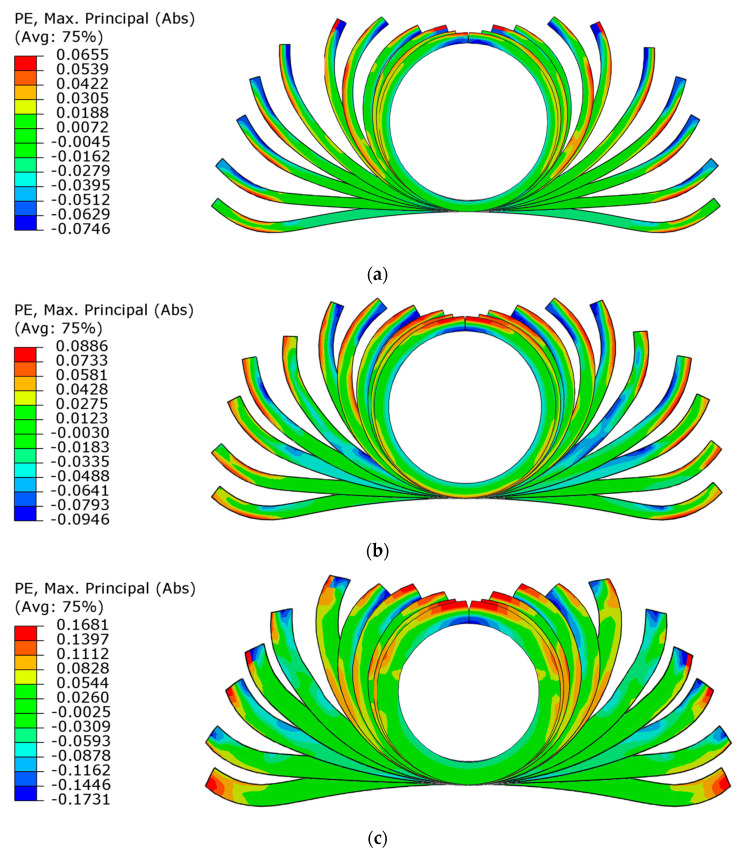
Plastic strain distribution of the flower pattern sections: (**a**) 3 mm; (**b**) 4 mm; and (**c**) 6 mm.

The plastic strain distribution on the flower pattern is shown in Figure 12. It can be seen that the inner side of the strip exhibits the compressive strain and the outer side exhibits tensile strain. Notably, the compressive strain is consistently slightly larger than the tensile strain, which may be attributed to the neutral axis offset. Halmos [32] summarized the neutral axis coefficient *k*, which can be expressed as follows:

(10) $$k=0.567\times \frac{\frac{R_{i}}{t}+0.25}{\frac{1.2R_{i}}{t}+1}\times \left(1+\frac{{R_{p0.2}}^{2.5}}{2051{R_{m}}^{1.41}}\right)$$

where *R_i_* is the inner radius, *t* is the wall thickness, and *R_m_* and *R*_*p*0.2_ are tensile strength and yield stress, respectively. The calculated *k* values corresponding to the wall thicknesses of 3, 4, and 6 mm are 0.55, 0.58, and 0.6, respectively, which exceed the value of 0.5. The results show that the neutral axis tends to shift outward after bending, causing an increase in the distance between the inner wall and the neutral axis, which induces a larger compressive strain.

The strain distributions of the strips with thicknesses of 3 and 4 mm are uniform during the roll forming process, meaning that the main deformation zone is evenly distributed along the forming arc. In contrast, for the 6 mm strip, the maximum strain is concentrated at the edges. With the increase in strip thickness, the maximum plastic strain increases continuously, which is consistent with the prediction of Equation (6). According to the flow stress curve shown in Figure 1, the maximum allowable plastic strain for the TA4 strip is 0.125, corresponding to a thickness of 4.6 mm, and exceeding this thickness poses a risk of cracking. Therefore, the plastic strain in the strips with thicknesses of 3 and 4 mm is located in the effective forming range, while the maximum plastic strain of the 6 mm thickness strip reaches 0.173, leading to edge cracking.

### 3.2. Effect of Roll Positioning

**Figure 13:**
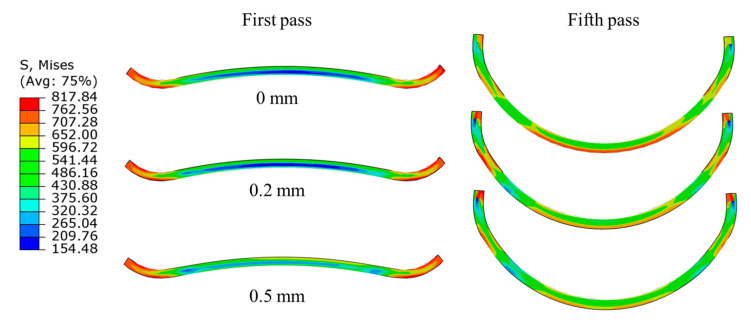
The Mises stress distributions varying with different forming gaps and passes.

**Figure 14:**
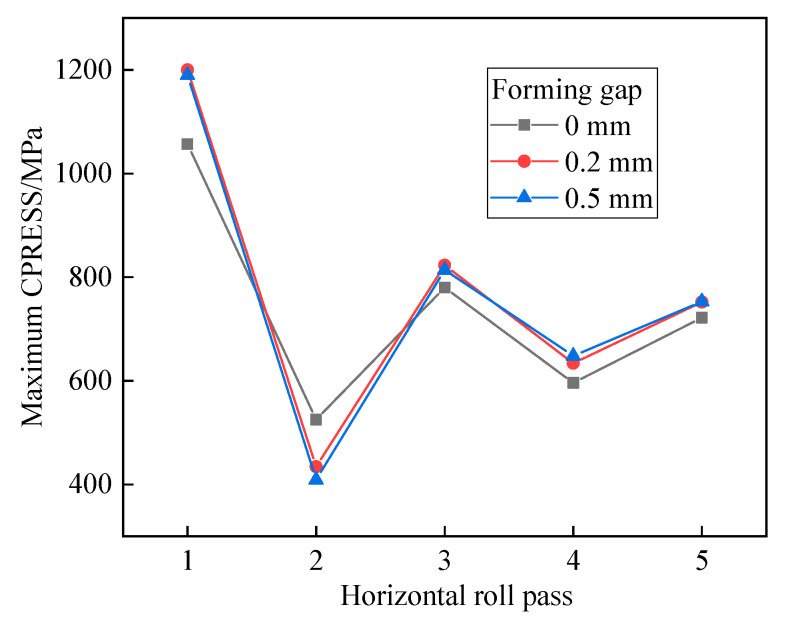
The maximum CPRESS of each pass roll.

Taking the horizontal rolls at the first and fifth passes as examples, we analyzed the influence of varying forming gaps on the Mises stress distribution, as illustrated in Figure 13. The results indicate that the forming gap has minimal impact on the stress distribution at the edge region during the first pass. However, in the fifth pass, as the forming gap increases, the region of maximum stress shifts from the central circular arc to the tangent point of the double radius arc and the edge of the strip. This shift indicates a change in the primary contact position between the strip and the rolls, leading to an increase in edge stress, which may adversely affect the tube formation process. The maximum contact pressure (CPRESS) for each pass is shown in Figure 14. It is evident that, except for the second pass through the horizontal rolls, the maximum CPRESS increases with the widening of the forming gap. This phenomenon occurs because an increased gap reduces the degree of fit, resulting in the forming force being concentrated over a smaller area. Therefore, it is crucial to keep the forming gap of the horizontal rolls as consistent as possible with the thickness of the tube in order to optimize the forming process.

**Figure 15:**
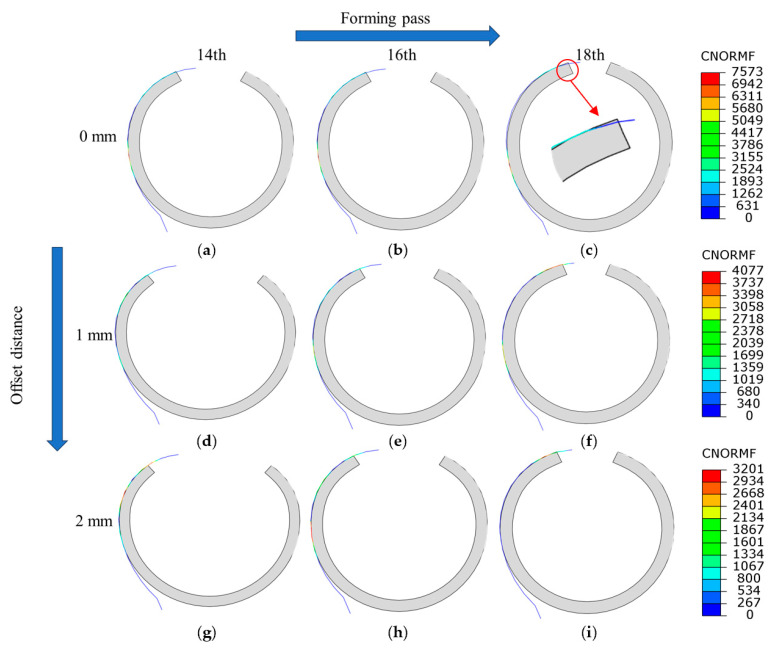
The contact state between the strip and vertical rolls: (**a**–**c**) 14–18 pass/0 mm; (**d**–**f**) 14–18 pass/1 mm; and (**g**–**i**) 14–18 pass/2 mm.

**Figure 16:**
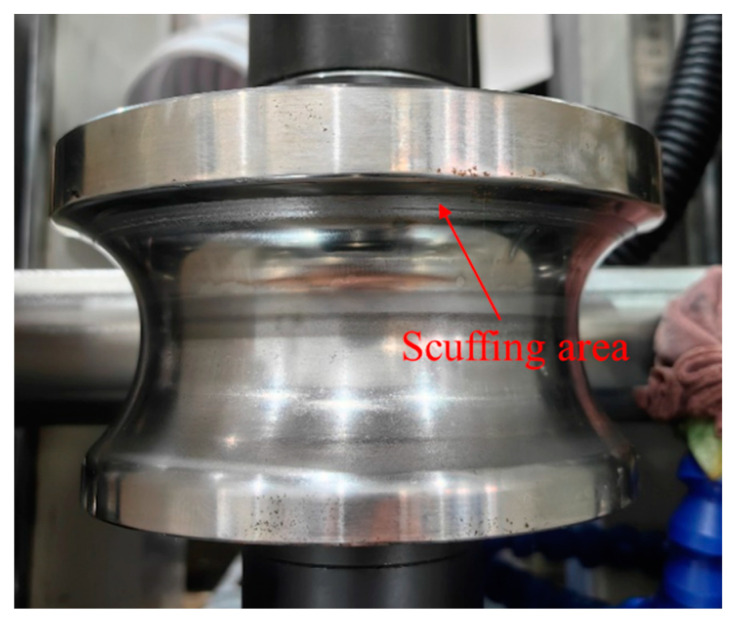
Scuffing position of vertical roll.

The rounding vertical rolls arranged at the positions of the 14, 16 and 18th passes, with varying contact diagrams between the strip and rolls, which are shown in Figure 15. The colored areas represent the contact positions. It can be seen from Figure 15a–c that when the vertical roll gap is 0 mm, the contact positions are primarily located at the edge and arc tangent point of strip, manifesting the point contact mode. Figure 15c shows a sharp edge contact with a maximum normal force of 7573 N, which risks roll surface scuffing or wearing, as confirmed by the actual scuffing position in Figure 16. When relaxing the rolls along both sides of the horizontal rolls by 1 mm, as seen in Figure 15d–f, the anastomosis degree between the strip and rolls was improved significantly, and the maximum normal force reduced to 4077 N. Further relaxing the vertical roll gap to 2 mm, the contact area continues to increase in the 14th and 16th passes (Figure 15g,h); however, in the 18th pass (Figure 15i), the roll contour surpasses the strip’s springback contour, leading to a return to edge contact. In summary, while aligning the roll tangent point so that it coincides with the strip tangent point is ideal, springback causes discrepancies, resulting in sharp contact and excessive pressure, which can be effectively improved by relaxing 1–2 mm of the vertical rolls basis.

**Figure 17:**
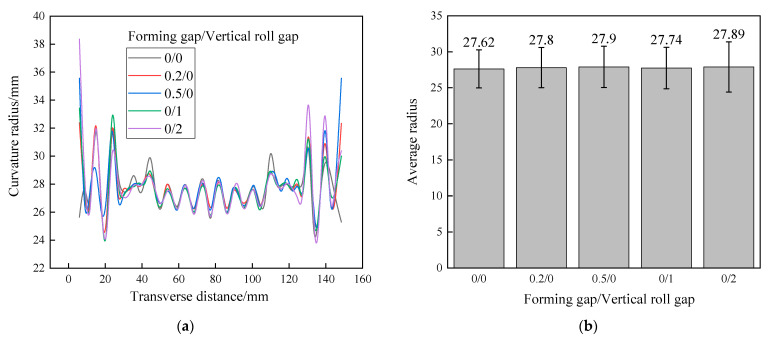
The outer radius in different roll positions: (**a**) radius along transverse; (**b**) average radius.

The radius of all tubes forming through different roll positions and after springback are illustrated in Figure 17. The radius distribution in the middle region of the tubes is relatively flat, but the edge region shows high local amplitudes due to the edge region only being formed into its target size in first roll pass. The average radiuses of tubes are almost the same, although the average radius increases slightly with the increase in the forming gap and the vertical roll gap, and the deviation value does not exceed 0.28 mm, which can be corrected in welding process. In addition, the final cross-section of the tube is always the same due to the fixed geometry of the last forming pass. Apart from the difference in stress distribution and contact state, there are no major differences in the tube geometry.

## 4. Roll Forming Experiment of TA4 Titanium CT

**Figure 18:**
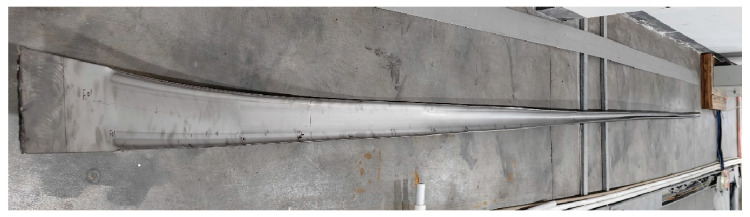
Trial production of TA4 titanium tube.

**Figure 19:**
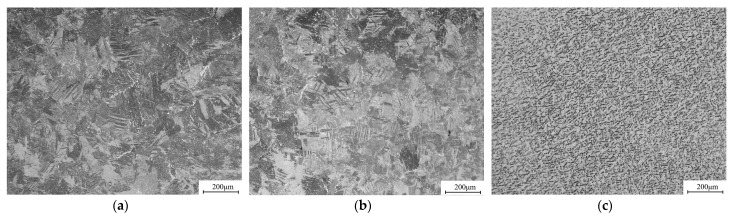
Microstructure of TA4 tube, 100×. (**a**) Welded zone; (**b**) heat affected zone; (**c**) base material.

The roll forming experiment was conducted on a roll forming production line using a TA4 strip with a thickness of 4 mm, a width of 149 mm and a length of 6 m. The target dimensions for the tube are an outer diameter of *d* = 50.8 mm and a thickness of *t* = 4 mm. The material properties of the strip are consistent with those described in Section 2.1. In the experiment, the W-forming strategy with the flower pattern and roll designed in Section 2.2 and Section 2.3 was adopted, the roll positioning parameters are the results optimized by numerical simulation. The strip sample was prepared across the whole forming process, from the first pass to the final pass, as shown in Figure 18. No defects, such as edge buckling, cracking and surface scuffing, were macroscopically observed during roll forming process. The microstructures of different zones of the tube are displayed in Figure 19; in addition, no defects like pores, inclusions and cracks were found in any of the zones. These results confirm that it is feasible to complete the roll forming of TA4 CT with the designed parameters.

**Figure 20:**
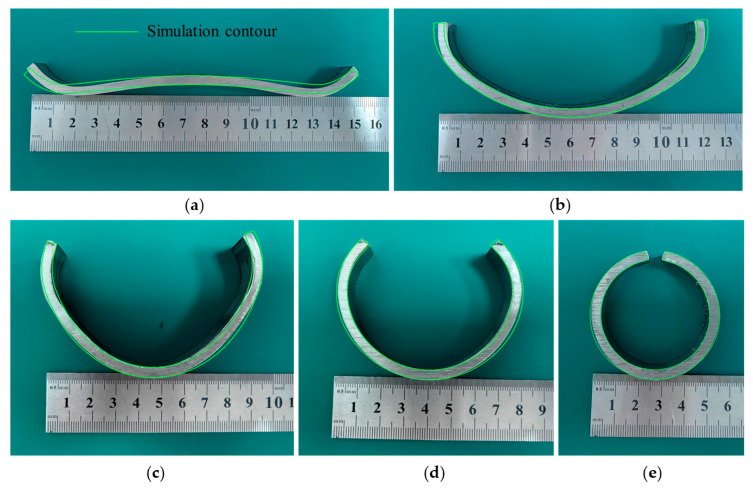
Experimental and simulation comparison of the roll forming: (**a**) pass 1; (**b**) pass 6; (**c**) pass 11; (**d**) pass 16; and (**e**) pass 21.

The cross-section comparison between the experimental and simulation results of the 1, 6, 11, 16, and 21st forming passes are shown in Figure 20. The simulation model significantly overestimates the springback of the material with a large bending radius, particularly as displayed in Figure 20a,b. This may be due to simulation modeling without considering the anisotropy of the strip. As the number of forming passes increases, the bending radius decreases, and the roll pass gradually ends, and the degree of correlation between the experimental and simulation results is improved.

**Figure 21:**
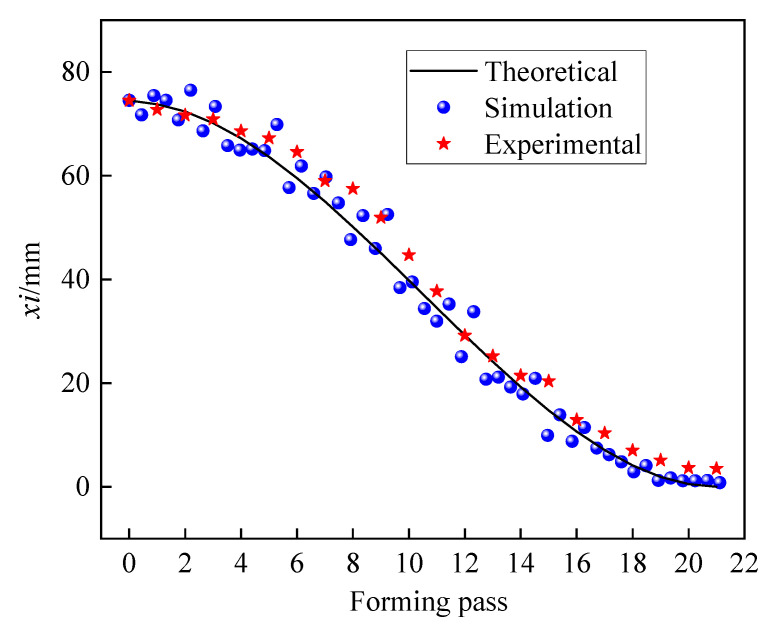
The verification of streamlined strip edge.

The variations in the strip edge projection distance *x_i_* with forming pass are shown in Figure 21. The streamlined theoretical forming conforms to a cubic function with the forming pass. It can be seen from the simulation results, influenced by springback and the dynamic explicit solver, that the scatter points exhibit slight fluctuations, but they remain closely aligned with the theoretical forming curve. Similarly, the experimental scatter points fall above the theoretical curve due to springback. In summary, the results of theoretical, simulation and experimental processes are consistent, which verified the accuracy of the established model.

## 5. Conclusions

In this paper, regarding the roll forming process of TA4 titanium with high strength and low elasticity, the flower pattern and roll design completed relied on the W-forming strategy and the cubic function of the strip edge projection. On this basis, the numerical simulation model was established in order to reveal the influence laws of tube dimensions and roll positioning. The main conclusions are as follows:

1. The formable TA4 titanium tubes should have a thickness/diameter ratio within a range of 0.051 to 0.092. A small ratio results in excessive springback, resulting in the strip not being caught smoothly in rolls, while a large ratio significantly increases the risk of edge cracking.
2. The roll positioning significantly affects the stress–strain distribution of the strip and its contact state but has little impact on the final geometry. According to the roll positioning optimization through numerical simulation, the forming gap should be matched to the thickness of the strip; the vertical roll gap should maintain a 1–2 mm clearance based on the location of the double radius arc tangent point between the strip and rolls.
3. The roll forming experiment conducted on the production line confirmed the feasibility of the optimized process parameters and successfully produced defect-free TA4 titanium tubes with a size of Φ50.8 × 4 mm. The results of theoretical analyses, numerical simulations, and the experiments are generally consistent. Incorporating the anisotropy of the strip in a subsequent study is expected to further improve the accuracy of the model.

## Figures and Tables

**Table 1:** Material properties of the TA4 titanium.

Density/g·cm^−3^	Elastic Modulus/GPa	Poisson’s Ratio	Yield Strength/MPa	Tensile Strength/MPa	Elongation/%
4.43	126	0.34	612	723	21

**Table 2:** The geometric sizes of each pass.

Pass	R_1_/mm	θ_1_/rad	*l*/mm	R_2_/mm	Pass	R_1_/mm	θ_1_/rad	R_2_/mm
1	23.4	0.94	7	−245	12	23.4	1.07	37
2	23.4	0.94	7	−570	13	23.4	1.20	34
3	23.4	0.94	/	550	14	23.4	1.20	32
4	23.4	0.94	/	172	15	23.4	1.20	30
5	23.4	0.94	/	115	16	23.4	1.28	28
6	23.4	1.07	/	96	17	23.4	1.28	26.5
7	23.4	1.07	/	75	18	23.4	1.28	25.4
8	23.4	1.07	/	62	19	23.4	1.57	24.9
9	23.4	1.07	/	53	20	23.4	1.57	24
10	23.4	1.07	/	46	21	23.4	1.57	23.4
11	23.4	1.07	/	41				

**Table 3:** Experiment parameters for numerical simulation.

Factors	Tube Dimensions/mm	Forming Gap/mm	Vertical Roll Gap/mm
A	50.8/2, 3, 4, 6	0	0
B	50.8/4	0, 0.2, 0.5	0
C	50.8/4	0	0, 1, 2

## Data Availability

The original contributions presented in the study are included in the article; further inquiries can be directed to the corresponding authors.
